# Temperature-induced oligomerization of polycyclic aromatic hydrocarbons at ambient and high pressures

**DOI:** 10.1038/s41598-017-08529-2

**Published:** 2017-08-11

**Authors:** Artem D. Chanyshev, Konstantin D. Litasov, Yoshihiro Furukawa, Konstantin A. Kokh, Anton F. Shatskiy

**Affiliations:** 1 V.S. Sobolev Institute of Geology and Mineralogy SB RAS, 3 Ac. Koptyuga ave., Novosibirsk, 630090 Russia; 2 Novosibirsk State University, 2 Pirogova st., Novosibirsk, 630090 Russia; 3 Department of Earth and Planetary Materials Science, Graduate School of Science, Tohoku University, Sendai, 980-8578 Japan

## Abstract

Temperature-induced oligomerization of polycyclic aromatic hydrocarbons (PAHs) was found at 500–773 K and ambient and high (3.5 GPa) pressures. The most intensive oligomerization at 1 bar and 3.5 GPa occurs at 740–823 K. PAH carbonization at high pressure is the final stage of oligomerization and occurs as a result of sequential oligomerization and polymerization of the starting material, caused by overlapping of π-orbitals, a decrease of intermolecular distances, and finally the dehydrogenation and polycondensation of benzene rings. Being important for building blocks of life, PAHs and their oligomers can be formed in the interior of the terrestrial planets with radii less than 2270 km.

## Introduction

High-pressure transformations of polycyclic aromatic hydrocarbons (PAHs) and benzene become extremely important due to wide applications for example in graphene- and graphene-based nanotechnology^1–3^, synthesis of organic superconductors^4, 5^, petroleum geoscience, origin of organic molecules in Universe and origin of life. In particular, PAHs were found in many space objects: meteorites^6–8^, cometary comae^9^, interstellar clouds and planetary nebulas^10–12^. Although the prevalent hypothesis for the formation of these PAHs is irradiation-driven polymerization of smaller hydrocarbons^13^, alternative explanation could be shock fragmentation of carbonaceous solid material^11^. PAH-bearing carbonaceous material could contribute to the delivery of extraterrestrial organic materials to the prebiotic Earth during the period of heavy bombardment of the inner Solar System from 4.5 to 3.8 Ga ago^14–16^.

PAHs are complex organic compounds consisting of condensed benzene rings. Pressure effect on the behavior of hydrocarbons has been intensively studied during recent years. The melting curves of naphthalene and benzophenone were determined at pressures up to 3 GPa using differential thermal analysis^17^. Limited temperature stability was defined for PAHs at 1.5–8.0 GPa^18–21^. Pressure-induced oligomerization/polymerization were found for benzene^22–24^, propene^25^, butadiene^26^, glycine^27, 28^ and alanine^28–30^. Benzene oligomerization was explained by the overlapping of π bonds and decrease of the intermolecular distances^23, 31^. Significant oligomerization of PAHs was observed from recovered samples after multi-anvil experiments at 7 GPa and 773–873 K^18^. Shock-wave experiments have revealed PAHs oligomerization and polycondensation at high pressure up to 30 GPa and estimated shock temperatures of 500–1660 K^32–34^.

In the past several decades, substantial understanding has been gained on the mechanism of PAHs thermal reactivity at ambient pressure. It was shown that that temperature effect on PAHs causes sequential coagulation, oxidation and soot formation^35–42^. Recent computational studies have revealed the nucleation mechanism of soot particles via covalent dimerization and oligomerization of PAHs^35, 36, 43^.

Here we investigated the oligomerization of several PAHs: naphthalene (C_10_H_8_), anthracene (C_14_H_10_), pyrene (C_16_H_10_), and coronene (C_24_H_12_) at ambient pressure and 3.5 GPa and high temperatures with application to the PAH abundance in cosmic bodies.

## Methods

All PAHs represent high-purity (99.9%) commercially available (Alfa-Aesar and Wako Co., Ltd.) crystalline solids. Experiments at ambient pressure and high temperature were performed in muffle furnace; the starting materials were naphthalene, anthracene and pyrene. Powder samples (40–60 mg) were loaded in the sealed quartz tubes. These experiments were performed at a saturated hydrocarbon vapor pressure, which is likely to be higher than 1 bar; however, the highest pressure value should not exceed ~20 bar, which is supposed to be a limit for quartz ampoules. For convenience, we assign the pressure values for these experiments as 1 bar. The samples were heated at a rate of 20 K/min; the exposure time at target temperature for these experiments was 30 min.

**Figure 1:**
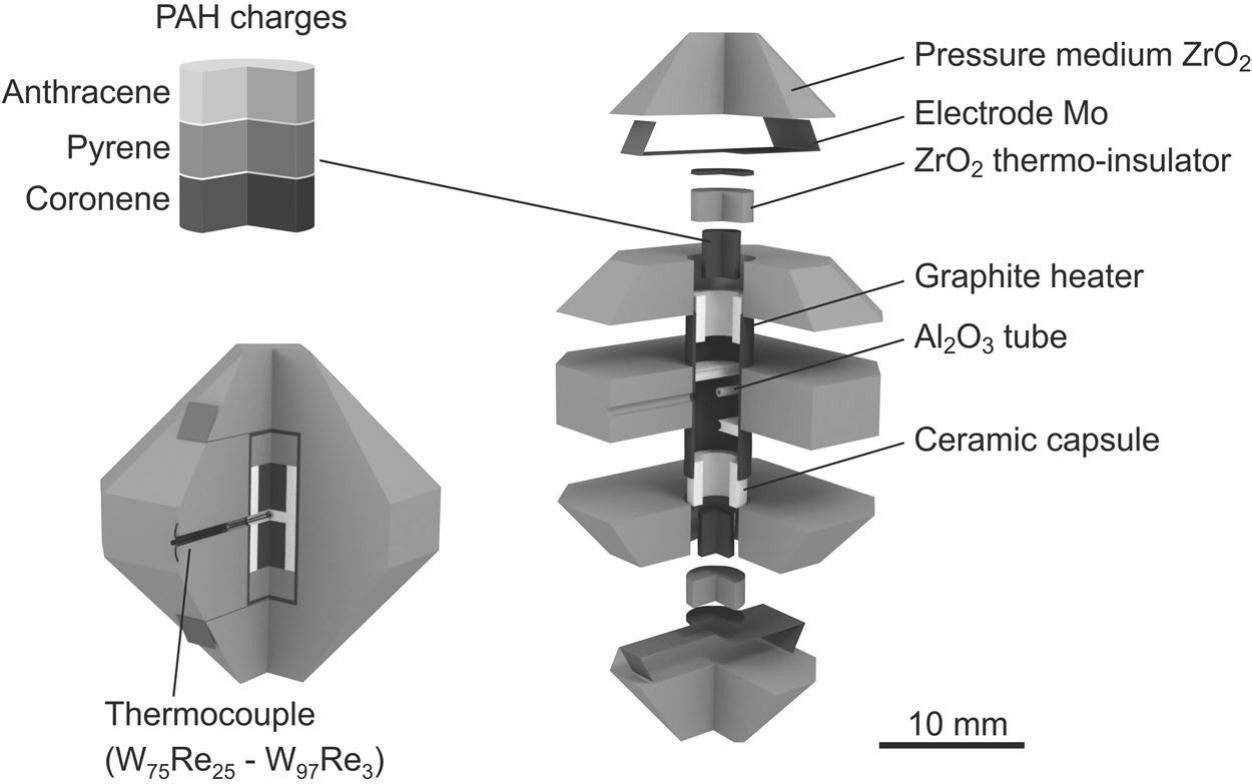
Schematic illustration of the high-pressure cell assemblage used for experiments.

Experiments at 3.5 GPa were performed using a Kawai-type 1500 tons multi-anvil apparatus at IGM SB RAS, Novosibirsk. We used WC anvils with a truncated edge length (TEL) of 12.0 mm. Pyrophyllite gaskets sealed the compressed volume and improve the stress distribution inside the anvils. ZrO_2_ semi-sintered ceramics (OZ-8C, MinoYogyo Co., Ltd) was used as a pressure medium, and a cylindrical graphite heater as the heating element (Fig. 1). The starting PAHs (naphthalene, anthracene and coronene) in the amount of 20–35 mg were inserted into the capsules from baked talc transformed to 3MgO·4SiO_2_ ceramics by heat treatment. The capsules included three isolated chargers with different PAHs. Temperature was monitored with a W_97_Re_3_ –W_75_Re_25_ thermocouple, inserted through the heater and electrically isolated by Al_2_O_3_ tubes. The lateral temperature variations across the charge did not exceed 10 °C, whereas the vertical temperature gradient was negligible. The detailed temperature measurement procedure is described in Litasov and Ohtani^44^.

The room-temperature pressure calibration was carried out by monitoring the resistance changes in Bi at 2.5 and 7.7 GPa^45^. The pressure calibration at high temperature was performed using known phase transitions in SiO_2_ (quartz-coesite)^46^ and CaGeO_3_^47^ at 1100 °C. The detailed pressure calibration procedures are described in previous studies^48, 49^.

The cell assembly was initially compressed to desired press load and then heated to the target temperature (500, 773 or 873 K) during several minutes and exposed for several hours (2–6 h).

After decompression, the extracted experimental products were studied by matrix-assisted laser desorption/ionization (MALDI) at Tohoku University. We used an AXIMA-CER Plus MALDI-TOF mass spectrometer (Shimadzu) to examine the PAH products. MALDI spectra were obtained using a linear time-of-flight (TOF) instrument. Neutral and ionized molecules were desorbed by a pulsed 337-nm ultraviolet nitrogen laser. The mass spectra of positive ions emitted directly in the desorption process were collected by the mass spectrometer at low laser power levels (10^6^ W/cm^2^). All spectra collected in this study are 300–1000 shot-averaged spectra. Sodium iodide standard was used to calibrate and test the MALDI system before sample analysis.

## Results and Discussion

**Figure 2:**
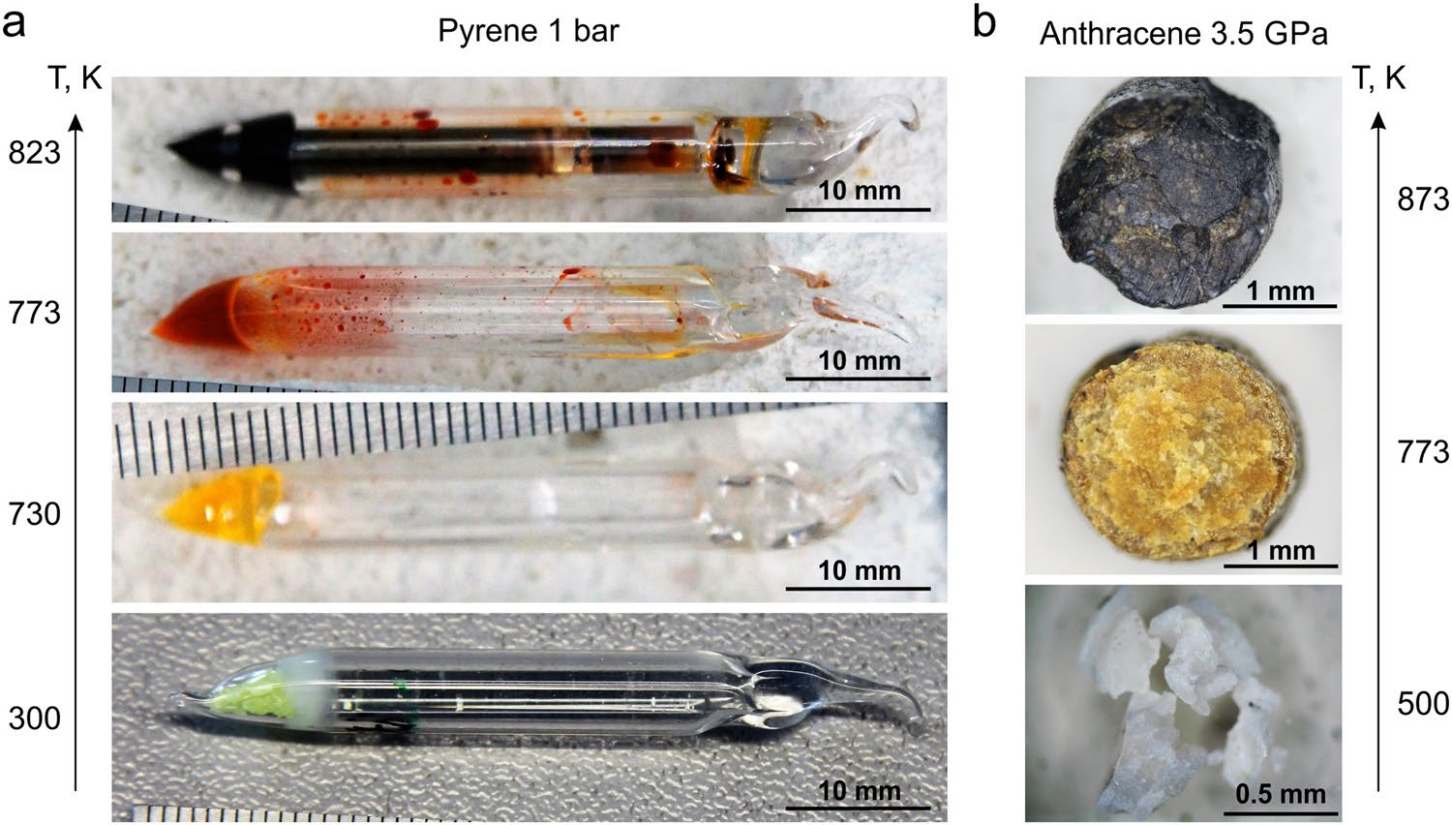
Images of the quenched samples after recovery. (**a**) – change in color of the pyrene reaction products at 1 bar and 300 (bottom image), 730, 773 and 823 (top image) K. (**b**) – change in color of the anthracene reaction products at 3.5 GPa and 500 (bottom image), 773 and 873 (top image) K.

At ambient pressure six experiments were performed at 700–845 K; at 3.5 GPa three experiments were performed at 500, 773 and 873 K. An important feature of PAH oligomerization is the gradual change of color from white or yellow through yellow, brown or red to dark brown and black (Fig. 2, Supplementary Fig. S1).

**Figure 3:**
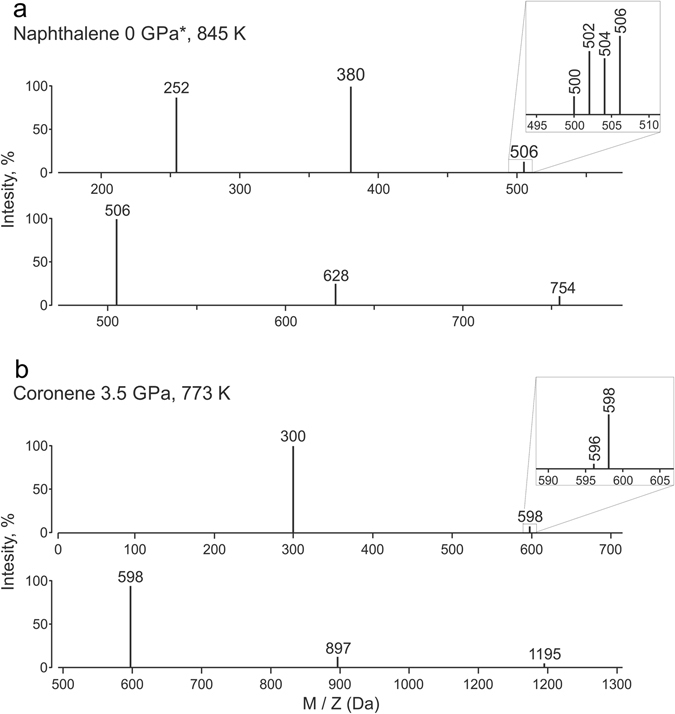
MALDI mass spectra of PAH experimental products. (**a**) – naphthalene at 1 bar and 845 K. (**b**) –coronene at 3.5 GPa and 773 K. *0 GPa = 1 bar.

The MALDI measurements revealed significant PAH oligomerization at ambient pressure and high temperature; the most intensive oligomerization (hexamer formation) was detected for naphthalene at 845 K (Fig. 3a), anthracene at 740 K and pyrene at 823 K (Table 1). Oligomerization temperature for anthracene is about 100 K less (706 K) than for naphthalene (820 K), pyrene (801) and coronene (800–820 K)^40^. Naphthalene (C_10_H_8_) and anthracene (C_14_H_10_) are polyacenes - PAHs consisting of linearly fused benzene rings. It was shown that polyacenes have higher aromaticity than non-linear PAHs (e.g. pyrene, coronene)^43^, since π electrons in polyacenes are concentrated around the central benzene ring that maximally increase its aromaticity^35, 36, 43^. DFT calculations clearly demonstrated that the propensity of dimer/oligomer formation increases with increasing length of linear polyacenes: anthracene dimer formation is energetically more favorable than the naphthalene dimerization^35, 36^.

**Table 1:** The results of MALDI analyses.

Compound	M, Da	P, GPa*	T, K	Dur., h	Capsule	Oligomerization**
Naphthalene, С_10_Н_8_	128.2	0.0	820	0.5	SiO_2_	635/5
		0.0	845	0.5	SiO_2_	754/6
Anthracene, С_14_Н_10_	178.2	0.0	700	0.5	SiO_2_	706/4
		0.0	740	0.5	SiO_2_	1052/6
		3.5	500	2.0	3MgO·4SiO_2_	178/1
		3.5	773	6.0	3MgO·4SiO_2_	710/4
		3.5	873	2.0	3MgO·4SiO_2_	355/2
Pyrene, С_16_Н_10_	202.3	0.0	773	0.5	SiO_2_	801/4
		0.0	823	0.5	SiO_2_	1195/6
		3.5	500	2.0	3MgO·4SiO_2_	202/1
		3.5	773	6.0	3MgO·4SiO_2_	999/5
		3.5	873	2.0	3MgO·4SiO_2_	1003/5
Coronene, С_24_Н_12_	300.4	3.5	500	2.0	3MgO·4SiO_2_	600/2
		3.5	773	6.0	3MgO·4SiO_2_	1195/4
		3.5	873	2.0	3MgO·4SiO_2_	599/2

*0.0 GPa = 1 bar.

**The numerator indicates the molecular weight of the largest determined oligomer with an intensity that exceed 1% of the maximum intensity; the denominator indicates the number of monomers presented in the oligomer.

At 3.5 GPa and 500 K the formation of coronene dimers was detected, whereas for anthracene and pyrene only starting material peaks were observed. At 3.5 GPa and 773 K we found anthracene oligomers up to 710 Da (tetramers), pyrene oligomers up to 999 Da (pentamers) and coronene oligomers up to 1195 Da (tetramers) (Fig. 3b). At 3.5 GPa and 873 K we observed formation of oligomers with atomic masses up to 355 Da (anthracene dimers), 1003 Da (pyrene pentamers) and 599 Da (coronene dimers). Therefore, the most intensive oligomerization at 3.5 GPa was found at 773 K. However the duration time for the experimental run of 3.5 GPa and 773 K was longer (6 h) than that for other runs at different temperatures (2 h). The kinetics of PAH reactivity at high pressure was experimentally studied in many works^50–52^. In particular, pressure increases the rate of PAH decomposition: the first-order decomposition rate constants of anthracene at 782 K were defined as *k*_1_ = 4.53 × 10^−4^ s^−1^ at 0.034 GPa and *k*_2_ = 2.53 × 10^−3^ s^−1^ at 0.2 GPa^52^. Pressure also increases the rate of PAH oligomerization. For anthracene at 763 K and 15 min, dimers formation was observed at 0.034 GPa, whereas tetramers were formed at 0.2 GPa^52^. Supplementary Table S1 lists the mass-spectrometric data for anthracene oligomerization products obtained by Whang *et al*.^52^ at 0.14 GPa and different temperatures and durations. It was clearly shown that at higher temperature (close to carbonization temperature) even with a longer duration, oligomerization of anthracene occurs less intensively (Table S1)^52^. Therefore one can suggest that exposure duration (exceeding few minutes) does not have a significant effect on the intensity of PAH oligomerization at high pressures.

Moreover, at high pressure the dimerization of polyacenes (anthracene) no longer favorable compared with non-linear PAHs (coronene). We attribute this difference to the features of the PAH oligomerization reaction at high pressures in solid state: the oligomerization reactions start at the boundaries between PAH crystals. Since the orientation of PAH crystals at high pressures can be arbitrary and the atoms are “rigidly” fixed in the crystal lattice (only thermal vibrations are permissible), the reaction interaction of solid anthracene molecules through the central benzene ring is difficult. The distribution of π electrons along the coronene molecule occurs evenly between all six outer benzene rings^43^; therefore, the aromaticity of coronene should not depend substantially on the aggregate form.

**Figure 4:**
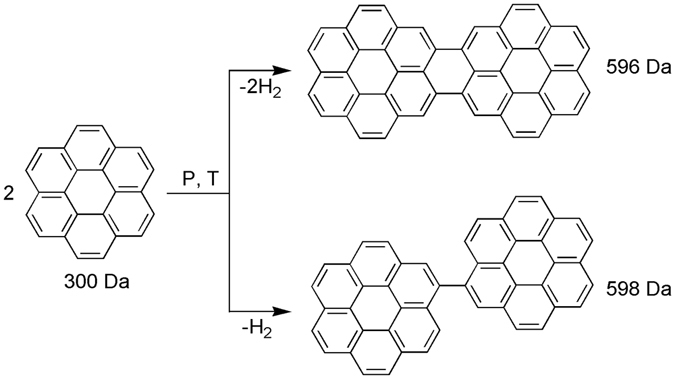
Dimerization of coronene at high pressure and temperature via dehydrogenation and formation of new carbon-carbon bonds.

PAH oligomerization at high pressures was carefully examined in recent experimental and theoretical studies^18, 22, 23^. It was argued that PAHs oligomer formation occurs via dehydrogenation and successive fusion of the initial hydrocarbon molecules through the C-C bond formation^18^. Alternatively, PAH compounds can be stacked by intermolecular forces. These stacked PAH compounds are called clusters^53, 54^. The molecular weights of PAH dimers stacked by intermolecular forces should be twice as heavy as the initial molecule. However in the present study the observed PAH compounds have lower molecular weights than PAH clusters; therefore here we observed formation of PAHs oligomers that consist of several aromatic units linked by C–C bonds (Fig. 4). For each oligomer, we observed several peaks within 5–10 Da, and the number of these peaks increased for higher oligomers (Fig. 3). The formation of these peaks could be explained via formation of single or several C–C bonds (Fig. 4).

A change in the color of the PAH reaction products with increasing temperature into the long-wavelength region (bathochromic shift) (Fig. 2, Supplementary Fig. S1) also supports the proposed mechanism. It was emphasized that with increasing size of the aromatic molecule, the HOMO-LUMO energy gaps (highest occupied molecular orbital - lowest unoccupied molecular orbital gap) is seen to decrease due to the condensation of higher-conjugated π-systems^55^, that leads to a bathochromic shift of the emitted light^56, 57^. Therefore one can suggest that the change in the color of the PAH reaction products is a consequence of the molecules size increasing, i.e. oligomerization.

The similar oligomer formation mechanism was found for alanine at pressures 9–11 GPa and 300 K, where alanylalanine and trialanine were formed via alanine dehydration and C-N bond formation^29^. Although we found PAH oligomerization at 3.5 GPa and 500–773 K, the average intermolecular distances of experimental products should be longer than that at a reaction threshold, because the diffraction patterns (and crystal structures) of these hydrocarbons do not undergo significant changes at selected parameters^20^.

**Figure 5:**
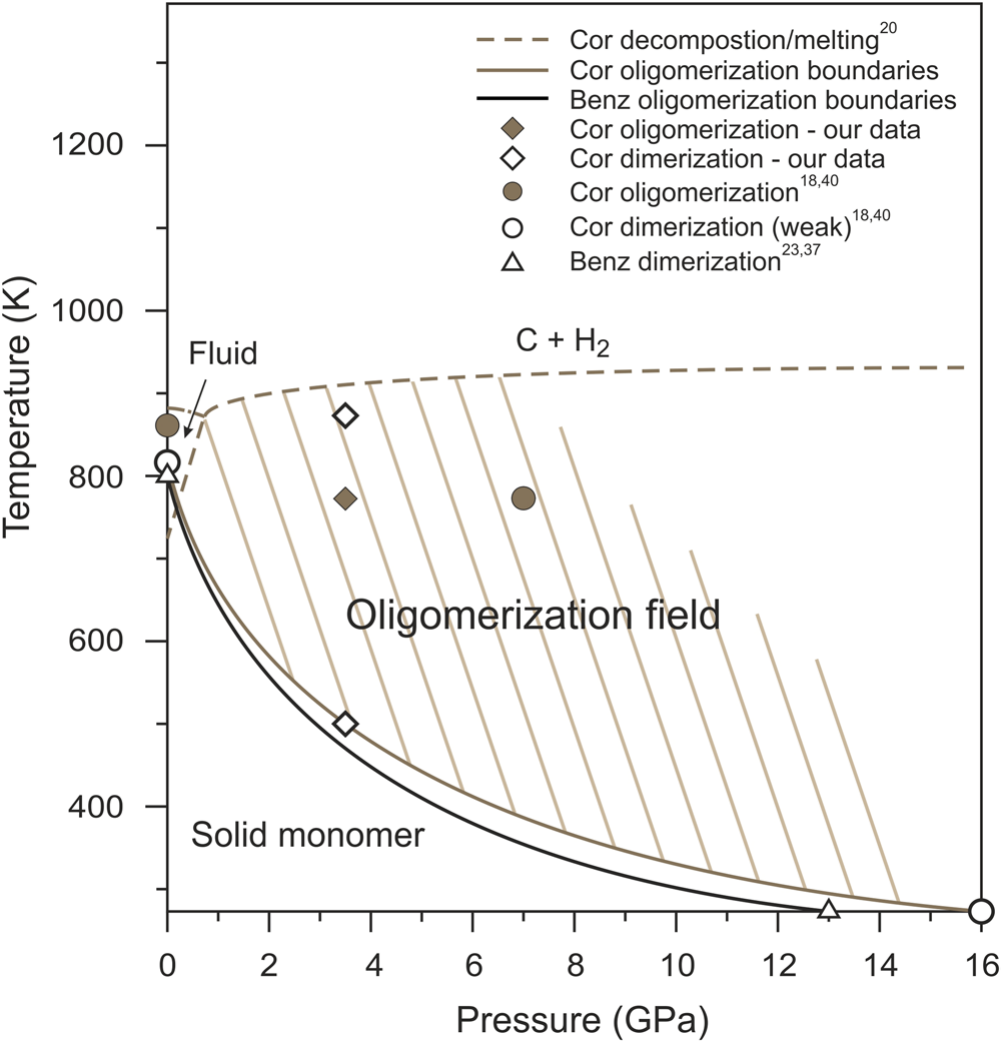
*PT*-diagram of coronene oligomerization. Diagram shows results in the timescale of 0.5–6 hours. Dimerization curves of coronene and benzene^23, 37^ are almost equals in the pressure range of 0–16 GPa. The shaded area corresponds to the coronene oligomerization field. Benz – benzene, Cor – coronene.

Based on the results of the MALDI analyzes of quenched experimental products (Table 1) and on the results of previous experimental studies at ambient^37, 40^ and high pressures^18, 22, 23^ we suggest *PT*-diagram of coronene oligomerization as well as benzene dimerization curve to 16 GPa and 1000 K (Fig. 5). Dimerization curves of coronene and benzene are almost equals in the pressure range of 0–16 GPa (Fig. 5). However, why does PAHs begin to oligomerize with increasing pressure at lower temperatures? Previously it was predicted that graphane-like polymers (C:H = 1:1) are more stable at high pressures than benzene^24^. Spontaneous conversion of benzene to polymers at low pressures (<20 GPa) does not occur due to significant kinetic barriers to polymerization^24^. At higher pressures ( > 20 GPa), benzene polymerizes at room temperature with the formation of carbon nanothreads^22, 58^. We suppose that in the pressure range 0–20 GPa, the conversion from benzene (as well as PAHs) to graphane-like polymers (four-coordinated C polymers) can occur under the influence of additional factors, such as exposure duration and elevated temperature. In particular, slow compression (several days) of coronene to 6 GPa at room temperature led to its amorphization^59^; in other studies, the crystalline coronene was detected at pressures to 17.1 GPa^19, 60^. The temperature increase at high pressures should also stimulate the PAH - polymer transition as well as dehydrogenation. We suppose that the simultaneous occurrence of these processes at high pressure should led to increase of the PAH aromaticity.

**Figure 6:**
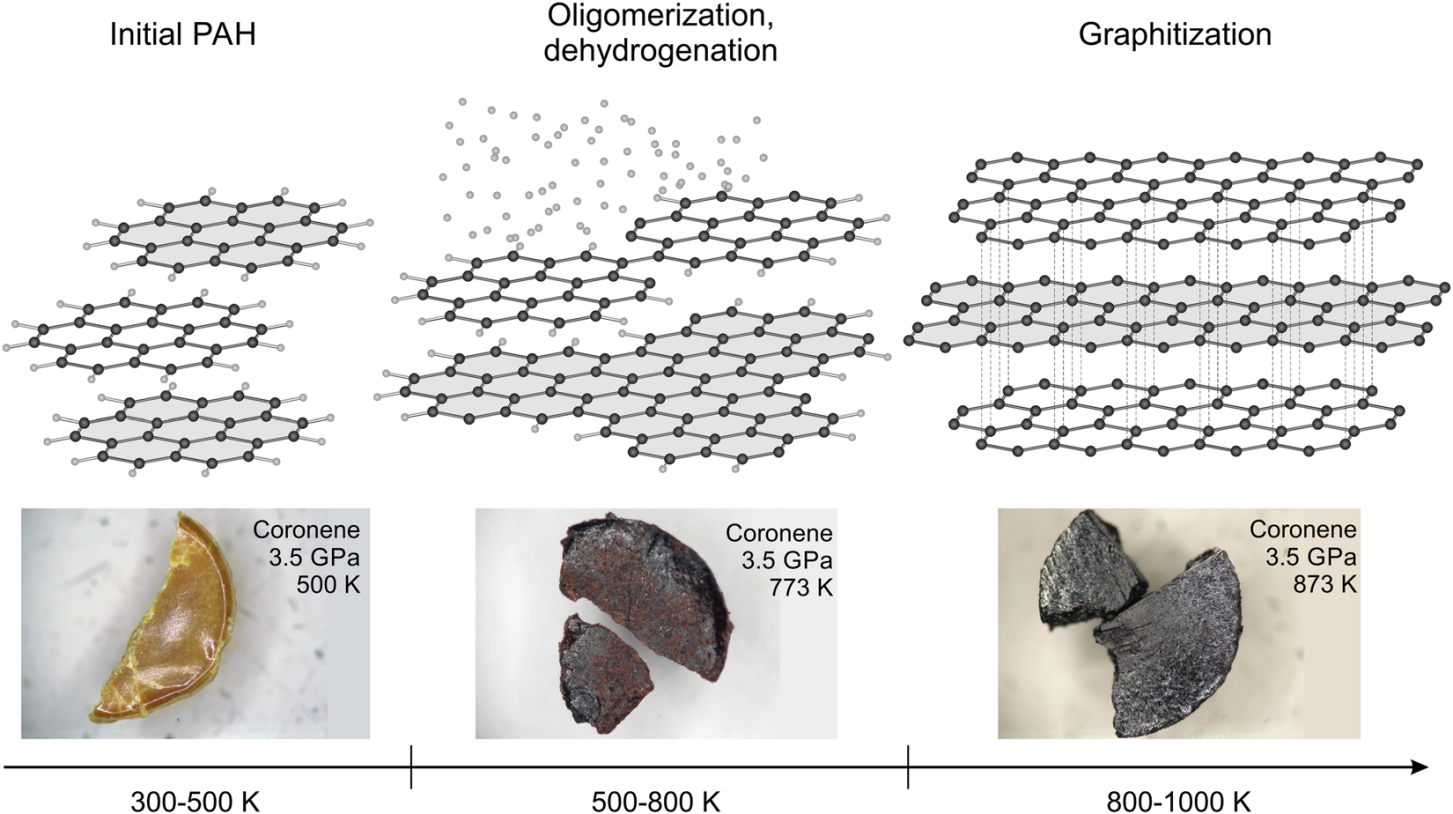
PAHs oligomerization and carbonization mechanism at high pressure and temperature. The steps for the PAHs carbonization can be divided into two stages: (1) PAH dehydrogenation and successive fusion of the initial hydrocarbon molecules through C-C bond formation (500–800 K) - change of color from white or yellow to yellow, red or brown; and (2) decrease of intermolecular distances and polycondensation of benzene rings (800–1000 K) - change of color from yellow, red or brown to dark brown or black.

Increasing temperature to 1000 K should lead to PAHs dissociation to carbon and presumably molecular hydrogen (or light hydrocarbon compounds)^18–21^. PAH carbonization occurs as a result of sequential oligomerization and polymerization of the starting material, caused by overlapping of π-orbitals, a decrease of intermolecular distances, and finally the dehydrogenation and polycondensation of benzene rings (Fig. 6). It was emphasized in the previous studies that the formation of oligomers simultaneously at high pressures and high temperatures was promoted by energetically activating hydrocarbons under influence of catalysts – copper or platinum^18, 61^. However in present study and in previous studies^23, 29^ oligomerization of organic compounds was observed at high pressures without any active catalyst.

PAH oligomerization at high pressures and high temperatures is extremely important for PAH chemistry in space and meteorites. PAHs found in meteorites^6–8^ could be a source of prebiotic organic matter in the early stages of the Earth formation. Shock-induced transformations and devolatilization of PAH were studied at 5.8–36.6 GPa and 500–1660 K^32, 33^. It was shown that PAHs in impactors survived only in the early stage of Earth and terrestrial planets formation, when it was <2270 km in radius^32^. Therefore, small planetoids of terrestrial type should be able to preserve PAHs and oligomers in their interiors.

In summary, our results demonstrated that at ambient pressure oligomerization of naphthalene, anthracene and pyrene occurs at 700–845 K; whereas at 3.5 GPa oligomerization of anthracene and pyrene was observed at 773–873 K and coronene at 500–873 K. The most intensive oligomerization at 1 bar and 3.5 GPa occurs at 773 K. Anthracene possesses higher aromaticity at ambient pressure in comparison with naphthalene and non-linear PAHs, whereas at 3.5 GPa coronene begins to oligomerize at lower temperatures in comparison with anthracene and pyrene. Oligomers are intermediate products of the PAH transformation into carbon (soot at ambient pressure, graphite at 3.5 GPa). In application, we emphasize PAHs and their oligomers survival in the interior of the terrestrial planets with radii less than 2270 km.

## Electronic supplementary material

Supplementary Materials
