# Supplementary material for: Temperature-induced oligomerization of polycyclic aromatic hydrocarbons at ambient and high pressures
Source: Sci Rep. 2017 Aug 11;7:7889. doi: 10.1038/s41598-017-08529-2 (PMC5554131; doi:10.1038/s41598-017-08529-2)
Supplement: Supplementary file 1 — Supplementary Materials [file 41598_2017_8529_MOESM1_ESM.pdf]

Title: Temperature-induced oligomerization of polycyclic aromatic hydrocarbons at ambient and high pressures.

Authors: Artem D. Chanyshev, Konstantin D. Litasov, Yoshihiro Furukawa, Konstantin A. Kokh & Anton F. Shatskiy

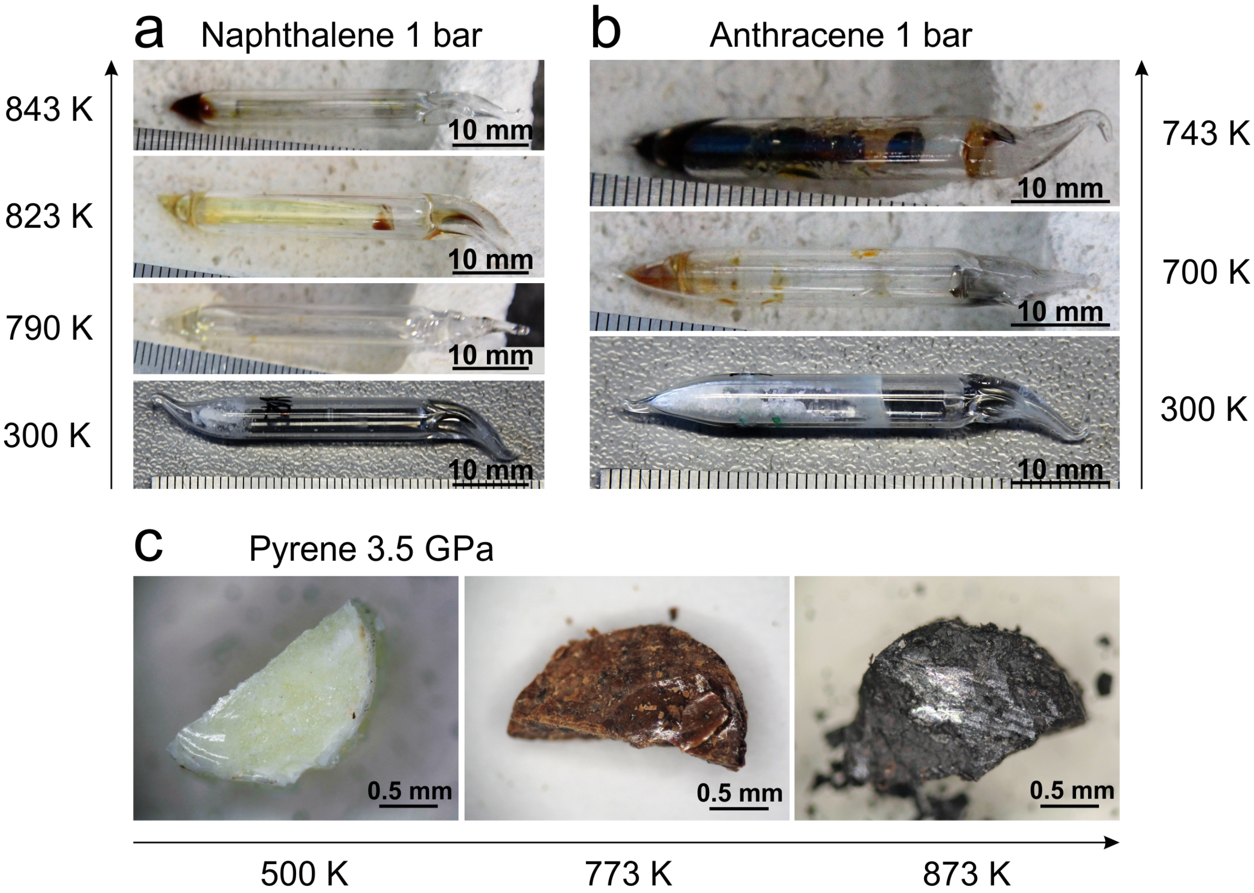

Figure S1. Images of the quenched samples after recovery. (a) – change in color of the naphthalene reaction products at 1 bar and 300 (bottom image), 790, 823 and 843 (top image) K. (b) – change in color of the anthracene reaction products at 1 bar and 300 (bottom image), 700 and 743 (top image) K. (c) – change in color of the pyrene reaction products at 3.5 GPa and 500 (left image), 773 and 873 (right image) K.

Table S1 – Mass spectrometric data from the solid resulting from the anthracene oligomerization at 0.14 GPa.

	M, Da	Relative intesity	
		763 K, 15 min	782 K, 60 min
monomer	174–180	vs	s
dimer	350–356	s	s
trimer	526–532	s	m
tetramer	700–715	m	?

Abbreviation: vs = very strong, s = strong, m = medium, ? – not detected.

Data are from Whang et al.[1]

References

1 Whang, P., Dachille, F. & Walker Jr, P. Pressure effects on the initial carbonization reactions of anthracene. *High Temp-High Press* **6**, 127-136 (1974).
